# Comparison of endoscopic submucosal dissection and transanal endoscopic surgery for the treatment of rectal neoplasia: A systematic review and meta-analysis

**DOI:** 10.1016/j.clinsp.2025.100613

**Published:** 2025-03-13

**Authors:** Igor Valdeir Gomes de Sousa, Alexandre Moraes Bestetti, Diego Paul Cadena-Aguirre, Angelo So Taa Kum, Paulo Ferreira Mega, Pedro Henrique Veras Ayres da Silva, Nelson Tomio Miyajima, Wanderley Marques Bernardo, Eduardo Guimarães Hourneaux de Moura

**Affiliations:** Gastrointestinal Endoscopy Service, Department of Gastroenterology, Hospital das Clínicas, Faculdade de Medicina da Universidade de São Paulo, São Paulo, SP, Brazil

**Keywords:** Rectal neoplasms, Transanal minimally invasive surgery, Endoscopic submucosal dissection, Transanal endoscopic microsurgery

## Abstract

**Background and aim:**

Minimally invasive techniques offer alternatives to conventional surgery in the treatment of early-stage colorectal cancer, reducing morbidity. Transanal Endoscopic Microsurgery (TEM) and Transanal Minimally Invasive Surgery (TAMIS) are widely used, while Endoscopic Submucosal Dissection (ESD) is gaining attention for its ability to achieve complete resection with low recurrence rates. This study compares the efficacy and safety of ESD with Transanal Endoscopic Surgery (TES).

**Methods:**

The authors performed a systematic review and meta-analysis of comparative studies involving patients with endoscopically resectable rectal lesions. Electronic searches were conducted in MEDLINE, EMBASE, Cochrane, and LILACS. Outcomes included recurrence rate, complete resection, en bloc resection, hospital stay, procedure time, and complication rate.

**Results:**

The analysis included ten observational studies and one Randomized Controlled Trial (RCT) involving 1,094 patients. No significant differences were found in terms of recurrence rate, en bloc resection, R0 resection, and complications between techniques. The RCT showed a shorter procedure time in the TES (RD = 16.6; 95 % CI 8.88 to 24.32; p < 0.0001), whereas observational studies found no significant difference. In addition, observational studies found a shorter hospital stay duration in the ESD (MD = -1.22; 95 % CI -2.11 to -0.33; I^2^ = 82 %; p < 0.007), while the RCT found no difference.

**Conclusion:**

ESD and TES are safe and effective for the treatment of early-stage rectal tumors. Rates of local recurrence, block resection, R0 resection, complications, and procedure time were similar. However, the RCT showed a shorter procedure time with TES, while observational studies showed a shorter hospital stay with ESD.

## Highlights

• Colorectal cancer is one of the most common neoplasms and a leading cause of cancer-related mortality worldwide.
• The treatment of colorectal neoplasia has evolved significantly over the past few decades due to advances in minimally invasive techniques.
• Although conventional surgery remains the gold standard for rectal tumors, ESD, TEM, and TAMIS emerged as effective and less invasive therapies for benign tumors and early cancers without nodal metastases, allowing lower morbidity.
• ESD and TES are safe and effective treatment options for early-stage rectal tumors.

## Introduction

Colorectal cancer is one of the most common neoplasms and the third leading cause of cancer-related mortality worldwide.^1^ With the development and advancement of screening programs, it is increasingly being diagnosed in its early stages, allowing resection using less invasive techniques. Early colorectal neoplasms are those confined to the mucosal and superficial submucosal layers (classified as Tis and T1 by the American Joint Committee on Cancer ‒ AJCC), without high-risk histopathologic features for lymph node metastasis.^2^

Conventional surgery for rectal tumors typically involves extensive tissue removal, which can lead to slower recovery, increased risk of complications, and significant changes in bowel function. On the other hand, minimally invasive techniques have emerged as an effective and less morbid alternative for the resection of these tumors. These techniques include ESD and TES.^2^, ^3^, ^4^

TEM and TAMIS are types of TES that allow for the minimally invasive treatment of rectal tumors. TEM is a technique that uses a rigid rectoscope for full thickness resection of tumors located 4 to 18 cm from the anal verge. The device has three working channels that allow the insertion of instruments. Insufflation is performed with a conventional carbon dioxide insufflator. Its use has some limitations due to the complex learning curve, high cost, and the risk of complications, including postoperative anorectal dysfunction.^5^, ^6^, ^7^ Developed in 2009, TAMIS has emerged as an alternative to TEM, offering a larger surgical field, a more accessible learning curve, and potentially lower cost. TAMIS uses a flexible disposable device that is inserted into the anal canal and also has three channels for the passage of laparoscopic instruments and a dedicated port for insufflation. This flexible device is shorter than the rigid rectoscope used in TEM and provides a wider working angle, which increases flexibility and facilitates instrument manipulation.^6^, ^7^, ^8^, ^9^, ^10^, ^11^, ^12^

ESD offers *en bloc* resection and high resection rates, especially for larger lesions. The technique involves injecting a hyperosmolar liquid solution into the submucosal layer, followed by an incision in the mucosal layer and dissection within the submucosal plane, between the muscularis propria and the superficial submucosa. This allows for proper management of blood vessels along the dissection path and enables the *en bloc* resection of lesions. ESD uses a single working channel in the endoscope, which can limit traction and device manipulation, making the removal of particularly complex lesions more difficult.^1^^,^^6^^,^^8^^,^^9^^,^^13^^,^^14^

Few studies compare these techniques, and there is no consensus on the best treatment for early rectal cancer. Therefore, the authors conducted this systematic review and meta-analysis to evaluate the efficacy and safety of these different treatment techniques for rectal tumors.

## Materials and methods

### Protocol and registration

This systematic review and meta-analysis was conducted in accordance with the recommendations of the Cochrane Handbook of Systematic Reviews of Interventions and the Preferred Reporting Items for Systematic Reviews and Meta-analysis (PRISMA).^15^ The trial protocol was registered in the International Prospective Register of Systematic Reviews (PROSPERO) under the accession number CRD42023494615.

### Eligibility criteria

The authors searched for clinical trials and comparative studies that evaluated patients with early-stage rectal epithelial tumors that could be resected using endoscopic methods or TES. There were no publication dates or language restrictions. Exclusion criteria were non-comparative studies, protocol phase studies, and studies that included subepithelial lesions. EMR was not included in this meta-analysis and was not considered comparable to TEM and TAMIS due to difficulties in monobloc resection of larger lesions (greater than 20 mm).

### Search strategy and information sources

The authors searched for articles in the following databases: MEDLINE, EMBASE, Cochrane, and LILACS. The search started in December 2023 and used the following “mesh terms” in MEDLINE: [(“Rectal neoplasm” OR “rectal neoplasms” OR “rectal neoplasia” OR “rectal neoplasias” OR “rectal tumor” OR “rectal tumors” OR “rectal cancer” OR “rectal cancers”) AND (“Dissection” OR “Dissections” OR “Endoscopic Mucosal Resection” OR “ESD” OR “Endoscopic Mucosal Resections” OR “Endoscopic Submucosal Dissection” OR “Endoscopic Submucosal Dissections”) AND (“Transanal endoscopic microsurgery” OR “Transanal endoscopic microsurgeries” OR “TEM” OR “Transanal endoscopic surgery” OR “Transanal endoscopic surgeries” OR “Transanal endoscopic surgical procedures” OR “Transanal endoscopic surgical procedures” OR “transanal minimally invasive surgery” OR “TAMIS OR Minimally invasive surgical procedures” OR “Minimally invasive surgery” OR “Endoscopic Mucous Membrane Resection” OR “endoscopic” OR “endoscopy”)].

### Study selection and data collection process

Two independent authors searched the database by accessing all records in the above sources by title. Potentially relevant studies were screened for eligibility using abstracts. If an abstract met the eligibility criteria or needed to be clarified, the full text was accessed. Duplicates were removed. Disagreements were resolved by mutual agreement and consultation with a third reviewer. Researchers used Excel spreadsheets to extract data and relevant results.

### Data items

The authors collected information on study characteristics from the included studies, such as author, year of publication, study design, and sample size (Table 1). Studies were selected based on prespecified inclusion and exclusion criteria. Outcomes analyzed included recurrence rate, R0 resection rate, en-bloc resection rate, perforation rate, bleeding rate, procedure time, and length of hospital stay. Not all of these outcomes were assessed in every study.

**Table 1:** Characteristics of the studies.

**Studies**	**Study design**	**Year**	**Patients**	**Intervention**	**Control**	**Outcomes**
Park et al.^14^	Observational	2012	63 patients with high-grade non-polypoid dysplasia or cancer invading the submucosa	ESD (30 patients)	TEM (33 patients)	Recurrence, en bloc resection, R0 resection, procedure time, hospital stay, bleeding, perforation
Kawaguti et al.^7^	Observational	2014	24 patients with early-stage rectal cancer	ESD (11 patients)	TEM (13 patients)	Recurrence, en bloc resection, procedure time, hospital stay
Tajika et al.^19^	Observational	2016	76 patients with low rectal tumor	ESD (48 patients)	TEM (28 patients)	Recurrence, en bloc resection, procedure time, hospital stay, complications
Mao et al.^20^	Observational	2017	57 patients with early-stage rectal tumor	ESD (31 patients)	CA-TAMIS (26 patients)	Recurrence, R0 resection, procedure time, bleeding, perforation
Mittal et al. (abstract)^21^	Observational	2018	50 patients with rectal polyps	ESD (31 patients)	TAMIS (patients)	Recurrence, en bloc resection, R0 resection, procedure time, bleeding, perforation.
Jung et al.^22^	Observational	2018	56 patients with epithelial rectal tumor	ESD (40 patients)	TEM (16 patients)	Recurrence, en bloc resection, R0 resection, procedure time, hospital stay, bleeding, perforation
Park et al.^23^	Observational	2021	285 patients with Neuroendocrine Tumors (NETs) measuring ≤2 cm	ESD (226 patients)	TEM (59 patients)	Recurrence, R0 resection, procedure time, hospital stay, complications
Kimura et al.^24^	Observational	2020	98 patients with adenomas and carcinoma	ESD (71 patients)	TEM (27 patients)	Recurrence, R0 resection, procedure time, hospital stay
Kim et al.^25^	Observational	2023	204 patients with early-stage rectal cancer	ESD (101 patients)	TEM (103 patients)	Recurrence, R0 resection, procedure time, hospital stay, complications
Jin et al.^26^	Observational	2023	114 patients with Neuroendocrine Tumors (NETs) measuring ≤2 cm	ESD (55 patients)	TEM (59 patients)	Recurrence, R0 resection, procedure time, hospital stay, complications
Yao et al.^27^	RCT	2019	67 patients with early-stage tumor	ESD (35 patients)	CA-TAMIS (32 patients)	Recurrence, R0 resection, bleeding, perforation, procedure time, hospital stay

Recurrence was defined as the return or resurgence of cancer following treatment. R0 resection was defined as neoplastic-free margins. En-bloc resection refers to the surgical removal of tissue or a tumor in one piece, preserving its integrity and continuity without fragmentation or dissection into smaller parts. Adverse events include any procedure-related event or incident, such as bleeding or perforation.

Length of hospital stay includes the entire hospital stay, from admission for the planned procedure to discharge from the hospital. Procedure time is the time from the insertion of the endoscope or the TEM or TAMIS device to complete the removal of the lesion.

### Risk of bias and quality of evidence

The risk of bias was analyzed using the Cochrane guidelines for adequate risk of bias exclusively for Non-randomized Interventional Studies (ROBINS-I),^16^ and Rob2 for randomized clinical trials (risk of bias tool for RCT).^17^ The quality of the evidence was graded into four levels: high, moderate, low, and very low, based on the objective criteria of the Grading of Recommendations Assessment, Development, and Evaluation process. This assessment was performed using the GRADE pro-Guideline Development Tool.^18^

### Statistical analysis

Data from the trials were collected and analyzed using Review Manager version 5.4 (RevMan 5.4) software. The authors collected data from each group for each outcome, expressed as absolute values, to calculate the risk difference between them. Risk differences were calculated using a fixed-effect model by the Mantel-Haenszel test for dichotomous outcomes, with a 95 % Confidence Interval (95 % CI). Values of p < 0.05 were considered statistically significant. Heterogeneity (inconsistency) was assessed and quantified using the Chi-Square test (χ^2^) and the Higgins method (I^2^). Heterogeneity (I^2^) values greater than 50 % were considered high, with a random-effects model chosen to evaluate this data, given the heterogeneity in the meta-analysis. For heterogeneity values less than 50 %, a fixed-effects model was employed. The McGrath method was used to convert the data to means and standard deviations for studies that reported results in medians and interquartile ranges.

## Results

### Overview

**Fig. 1:**
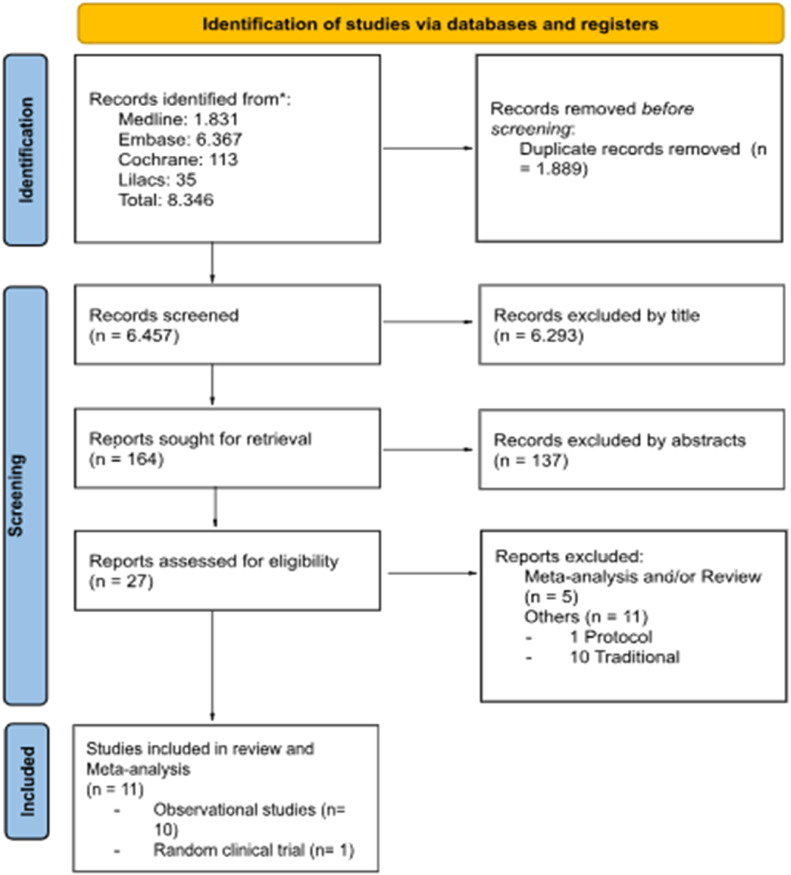
PRISMA flow diagram.

A total of 1831 articles were identified through a search in the PubMed database. An additional 6515 articles were identified in the EMBASE, Cochrane, and LILACS databases. After excluding duplicate articles and applying eligibility criteria, ten retrospective cohort studies and one randomized clinical trial remained (Fig. 1).

Ten included studies were observational studies^7^^,^^14^^,^^19^, ^20^, ^21^, ^22^, ^23^, ^24^, ^25^, ^26^ and one randomized controlled trial.^27^ Two observational studies compared ESD and TAMIS, while the other eight compared ESD and TEM. These eleven studies evaluated 1094 patients: 679 in the ESD group and 415 in the TES group (Table 1).

### Risk of the bias and quality of the evidence

**Fig. 2:**
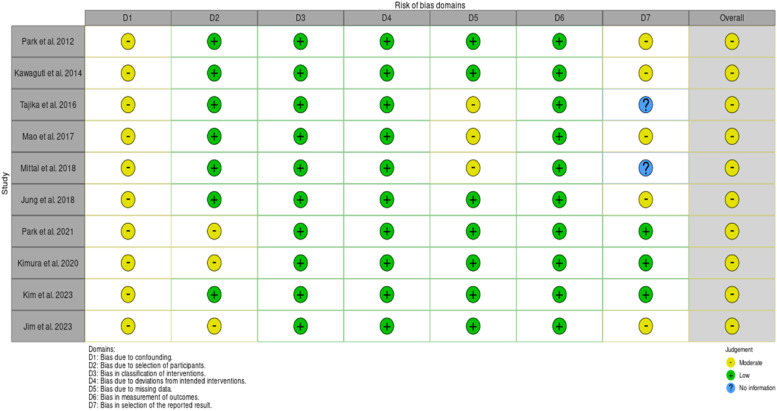
Risk of bias assessment assessed by ROBINS-I.

**Fig. 3:**
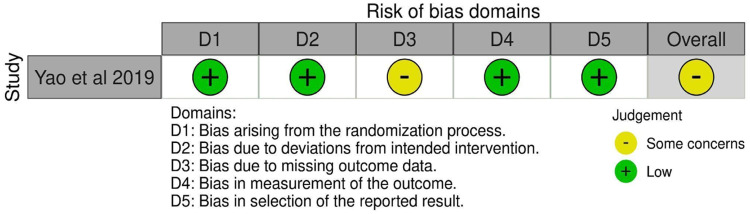
Risk of bias assessment assessed by Rob2.

**Fig. 4:**
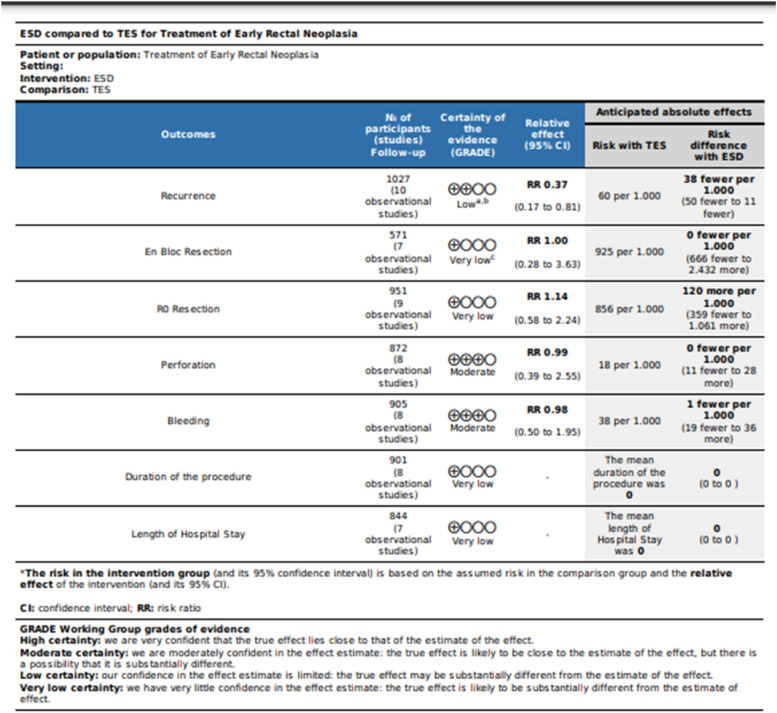
GRADEpro – Observational studies. Assessment of the quality of evidence for the comparison of ESD vs. TES for the treatment of early rectal neoplasia.

**Fig. 5:**
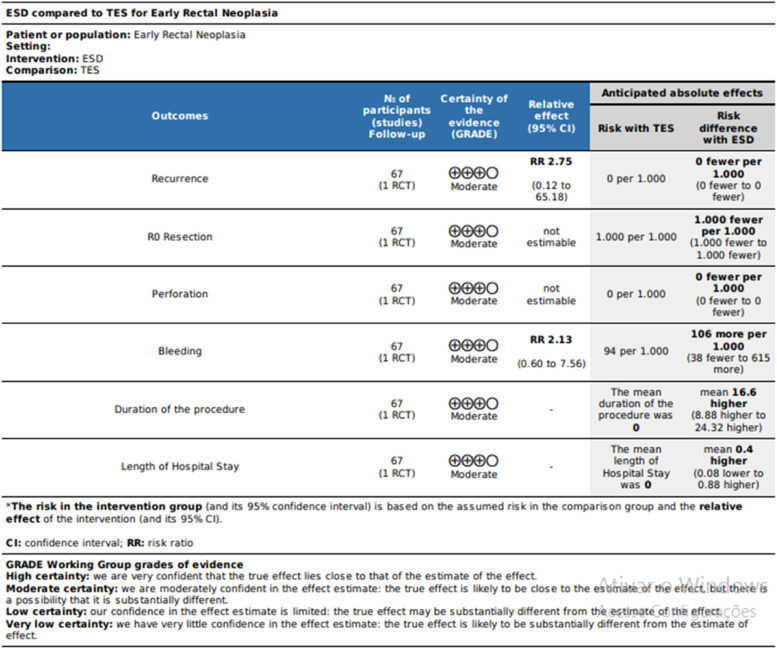
GRADEpro – RCT. Assessment of the quality of evidence for the comparison of ESD vs. TES for the treatment of early rectal neoplasia.

Using the Robins-I tool to assess the observational studies, the authors found that all had a moderate risk of bias (Fig. 2), and using the Rob2 tool to assess the RCT, the authors found some concerns (Fig. 3). In the observational studies, the GRADE-assessed quality of evidence was very low for *en bloc* resection, R0 resection, procedure time, and length of hospital stay; was low for recurrence and moderate for perforation and bleeding (Fig. 4). According to GRADE, the quality of evidence for all outcomes was moderate for RCT (Fig. 5).

### Outcomes

#### Recurrence

**Fig. 6:**
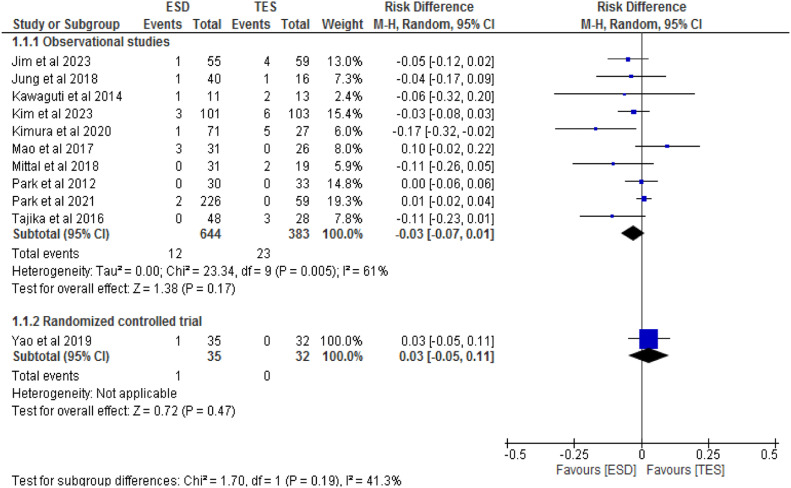
Forest plot recurrence rate.

All eleven studies, consisting of ten observational studies[7,14,19–26] and one RCT,^27^ totaling 1094 patients, compared recurrence rates between the ESD (679 patients) and TES (415 patients) groups. In the observational studies, there was no significant difference between the methods (RD = -0.03; 95 % CI -0.07 to 0.01; I^2^ = 61 %; p = 0.17). The RCT also found no significant difference between the groups (RD = 0.03; 95 % CI -0.05 to 0.11; p = 0.47), as shown in Fig. 6.

#### En bloc resection

**Fig. 7:**
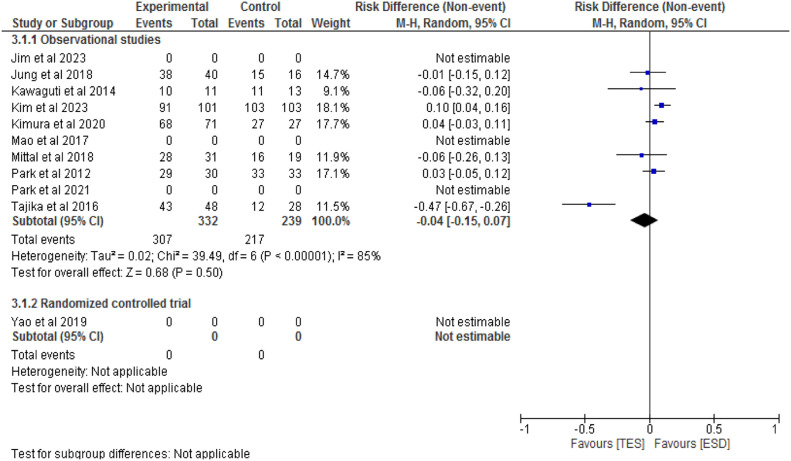
Forest plot en bloc resection rate.

Seven observational studies[7,14,19,21,22,24,25] with a total of 571 patients have compared en bloc resection rates between ESD (332 patients) and TES (239 patients). There is no significant difference between the methods (RD = -0.04; 95 % CI -0.15 to 0.07; I^2^ = 85 %; p = 0.5), as shown in Fig. 7. The RCT did not evaluate this outcome.

### R0 resection

**Fig. 8:**
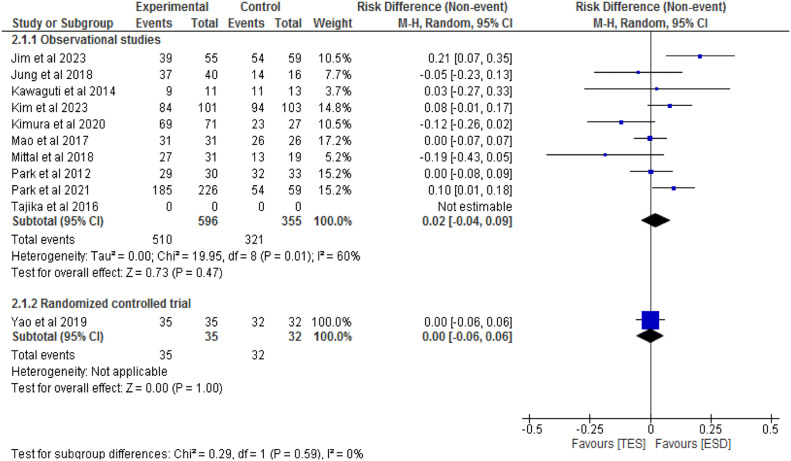
Forest plot R0 resection rate.

Ten studies, consisting of 9 observational studies[7,14,20–26] and one RCT,^27^ totaling 1018 patients, compared the rates of R0 resection between the ESD (631 patients) and TES (387 patients) groups. Among observational studies, there was no significant difference between the methods (RD = 0.02; 95 % CI -0.04 to 0.09; I^2^ = 60 %; p = 0.47). The RCT also found no significant difference between the groups (RD = 0; 95 % CI -0.06 to 0.06; p = 1), as shown in Fig. 8.

#### Perforation

**Fig. 9:**
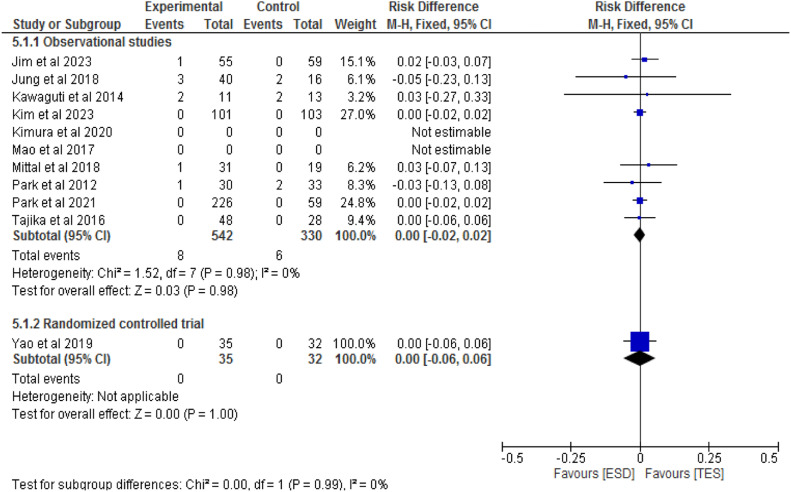
Forest plot perforation rate.

Nine studies, including eight observational studies[7,14,19,21–23,25,26] and one RCT,^27^ totaling 939 patients, compared perforation rates between ESD (577 patients) and TES (362 patients). In the observational studies, there was no significant difference between the methods (RD = 0; 95 % CI -0.02 to 0.02; I^2^ = 0 %; p = 0.98). The RCT also found no significant difference between the groups (RD = 0; 95 % CI -0.06 to 0.06; p = 1), as shown in Fig. 9.

#### Bleeding

**Fig. 10:**
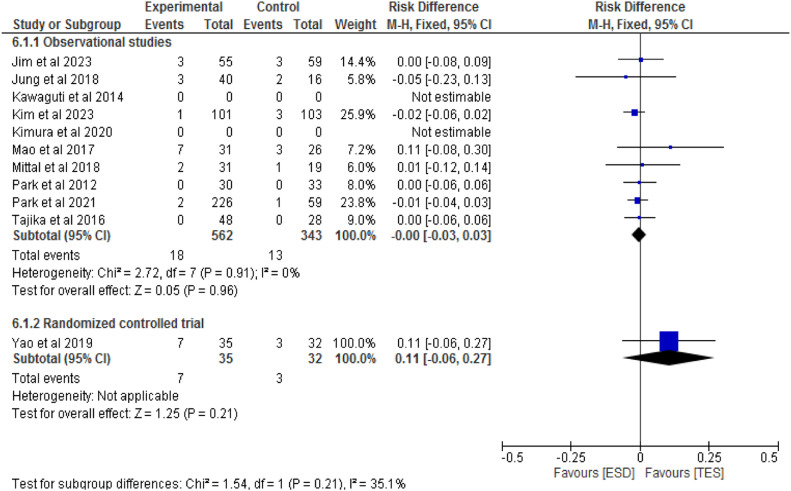
Forest plot bleeding rate.

Nine studies, including eight observational studies^14^^,^^19^, ^20^, ^21^, ^22^, ^23^^,^^25^^,^^26^ and one RCT,^27^ totaling 972 patients, compared bleeding rates between ESD (612 patients) and TES (394 patients). In the observational studies, there was no significant difference between the methods (RD = 0; 95 % CI -0.03 to 0.03; I^2^ = 0 %; p = 0.96). In the RCT, there was also no significant difference between the groups (RD = 0.11; 95 % CI -0.06 to 0.27; p = 0.21), as shown in Fig. 10.

#### Duration of the procedure

**Fig. 11:**
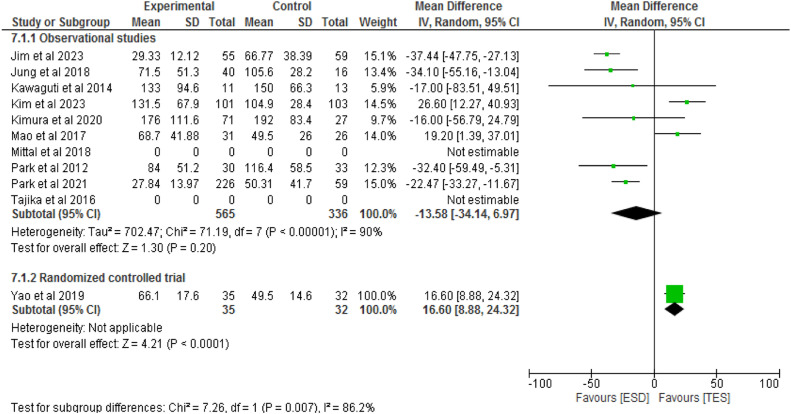
Forest plot procedure time.

Nine studies, including eight observational studies^7^^,^^14^^,^^20^^,^^22^, ^23^, ^24^, ^25^, ^26^ and one RCT[27] totaling 968 patients, compared the procedure time between ESD (647 patients) and TES (426 patients). In the observational studies, there was no significant difference between the methods (RD = -13.58; 95 % CI -34.14 to 6.97; I^2^ = 90 %; p = 0.2). The RCT showed a significant difference between the groups (RD = 16.6; 95 % CI 8.88 to 24.32; p < 0.0001), as shown in Fig. 11.

#### Length of Hospital Stay

**Fig. 12:**
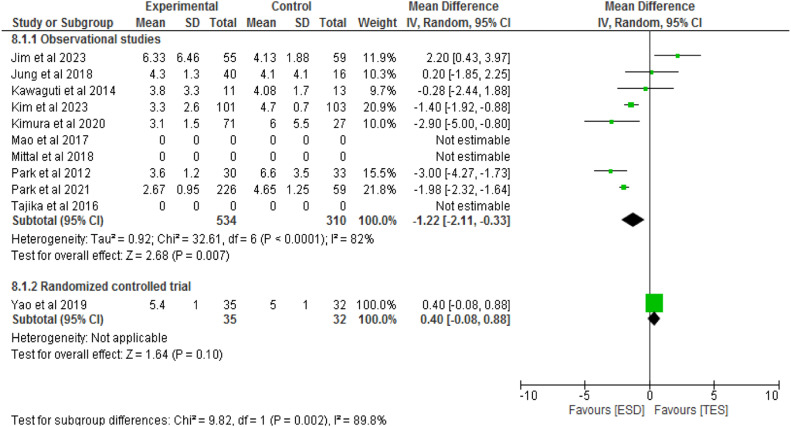
Forest plot length of hospital stay.

Eight studies, consisting of seven observational studies[7,14,22–26] and one RCT[27] totaling 911 patients, compared the length of hospital stay between ESD (682 patients) and TES (458 patients). In the observational studies, there was a significant difference between the methods (MD = -1.22; 95 % CI -2.11 to -0.33; I^2^ = 82 %; p < 0.007). In the RCT, there was no significant difference between the groups (RD = 0.4; 95 % CI -0.08 to 0.88; p = 0.1), as shown in Fig. 12.

## Discussion

The treatment of colorectal neoplasia has evolved significantly over the past few decades due to advances in minimally invasive techniques. Although conventional surgery remains the gold standard for rectal tumors, ESD, TEM, and TAMIS emerged as effective and less invasive therapies for benign tumors and early cancers without nodal metastases, allowing lower morbidity. Previous meta-analyses did not include recently published studies, including a randomized clinical trial, addressing endoscopic methods technical development.^25^^,^^28^

This is the largest meta-analysis to date evaluating ESD versus TES for early rectal neoplasia. The authors evaluated several recently published comparative studies, including more patients than previous meta-analyses.^6^^,^^29^^,^^30^ The present study did not find a significant difference between the observational studies and the RCT regarding the recurrence rate, R0 resection, perforation, and bleeding. However, procedure time was dichotomized. While ESD and TES had no significant difference between the observational studies, the RCT by Yao et al. showed a shorter procedure time in the TES group. These results may have been influenced by the high heterogeneity of the observational studies (I^2^ = 90 %) and the small number of patients in the RCT. Another dichotomous outcome was the length of hospital stay. While observational studies showed a shorter length of stay in the ESD group, the RCT by Yao et al. did not reach statistical significance. Although statistical significance was not reached for some outcomes, this analysis showed a favorable trend for the ESD technique.

These findings differ somewhat from those of previously published studies. Arezzo et al.^29^ in 2014 evaluated a case series comparing ESD (11 patients) and TEM (10 patients): TEM had higher rates of en bloc resection and R0 resection, as well as shorter procedure times, although the incidence of complications was similar between ESD and TEM. These discrepancies are likely because colorectal ESD is a more recently developed technique.

Unlike the study by Sagae et al. 2020,^6^ the authors found a significant reduction in the length of hospital stay in the ESD group. It is important to note that the authors included more studies, including recent publications and evaluations of different TES methods. A systematic review by McCarty et al. in 2020,^30^ which included only three studies, found that procedure time was shorter in the ESD group. However, the present study, which included more studies, did not find a significant difference in procedure time, demonstrating the similarity of methods for this outcome. With technical developments, better equipment, and improvements in recent years, the conditions for the procedures have improved, which may explain the difference in these outcomes.

In addition, the studies by Mittal et al.^21^ and Tajika et al.^19^ are only available as abstracts and lack details on inclusion criteria and patient follow-up periods. The studies by Mao et al.^20^ and Yao et al.^27^ compared ESD with a minimally invasive transanal surgery technique using a Glove Port (CA-TAMIS-GP). This method uses a glove as a portal attached to the anoscope, a colonoscope for optics, illumination, insufflation, and suction, and laparoscopic forceps for resection. In the study by Jung et al.,^22^ the lesions were categorized as epithelial and subepithelial. The authors chose to analyze only the data from the first group because the indication for ESD in subepithelial tumors is controversial.

Yao et al.^27^ emphasize that CA-TAMIS has the advantage of lower intraoperative bleeding, with statistical significance, possibly due to the more precise hemostatic effect of the ultrasonic scalpel used in this technique. Although the authors could not assess the cost difference between the modalities, Yao et al. highlight that the costs associated with CA-TAMIS were lower than those of ESD. This finding suggests that this technology has significant advantages in terms of accessibility and economics.

Considering only studies published in the last 5-years (2018‒2023), ESD has shown a higher rate of en bloc resection with statistical significance, highlighting advances in procedures and the technical proficiency of endoscopists, allowing for better outcomes in ESD (Supplementary Fig. 1).

This study has some limitations. Firstly, 10 of the included studies are observational, with significant drawbacks such as patient selection and information bias. Second, the groups were not homogeneous among the observational studies, although they were similar within each study. Third, there were differences in follow-up time, characteristics of resected lesions, and specific techniques used. In addition, the procedures were performed by professionals with different levels of experience and different learning curves. TEM and TAMIS are performed by surgeons, while ESD is performed by endoscopists. The choice between these methods depends on local availability and expertise, which, together with the technical differences mentioned above, may limit direct comparisons between them.

Despite these limitations, it is essential to emphasize that this review has significant value for several reasons, including providing essential guidance to assist professionals in clinical decision-making when dealing with early-stage rectal tumors. In addition, the authors included 5 recently published studies with a substantial number of patients, resulting in more reliable cumulative effects than previous meta-analyses. Finally, these limitations could be overcome by a comprehensive study evaluating all comparative studies of randomized clinical trials, with a low risk of bias. The TRIASSIC trial, a multicenter randomized clinical trial, will provide additional evidence to improve the understanding of this issue.^10^

Of the 7 outcomes assessed in the present study, 4 had a very low level of evidence according to the GRADE assessment for observational studies. The lower level of evidence is primarily due to the nature of the observational studies and the heterogeneity, which was greater than 50 % in 5 and greater than 75 % in 3 of the outcomes evaluated. The use of a random effect is important to control for high heterogeneity.

In addition, since this is a meta-analysis that included studies published in the last 12 years, it is understandable that with more experience with the procedure, there is a progressive change in its results. Moreover, the different centers performing the procedures have their own protocols regarding the procedure, care, and hospitalization, which could explain the high heterogeneity found.

In summary, the present results demonstrate that ESD and TES are safe and effective options for treating early-stage rectal tumors. Furthermore, given this similarity and a trend toward shorter hospital stays, ESD treatment provides optimal preservation of quality of life and rapid recovery compared to TES. Therefore, both procedures can be performed in patients with early rectal tumors, and the best approach should be individualized, considering personal and local expertise as well as the availability of materials and equipment. However, due to the limitations of the included observational studies, it is crucial to continue research and conduct randomized clinical trials to consolidate these findings and improve clinical practice in the treatment of early rectal tumors.

## Conclusion

Although there is a very low level of evidence for several outcomes assessed in this study, the authors conclude that ESD and TES are safe and effective treatment options for early-stage rectal tumors. Comparisons between randomized and observational studies have yielded divergent results regarding procedure time and hospital length of stay. The RCT showed a shorter procedure time in the TES group, while observational studies did not find a significant difference. In addition, observational studies found a shorter hospital stay duration in the ESD group, while the RCT found no difference.

## Funding

None.

## Declaration of competing interest

The authors declare no conflicts of interest.

## Appendix Supplementary materials

Image, application 1
