# Supplementary material for: Comparison of endoscopic submucosal dissection and transanal endoscopic surgery for the treatment of rectal neoplasia: A systematic review and meta-analysis
Source: Clinics (Sao Paulo). 2025 Mar 13;80:100613. doi: 10.1016/j.clinsp.2025.100613 (PMC11957494; doi:10.1016/j.clinsp.2025.100613)

**CLINICS-D-24-00711_ Supplementary Material**

**Supplementary Figure 1** Forest Plot en bloc resection considering studies published since 2018.


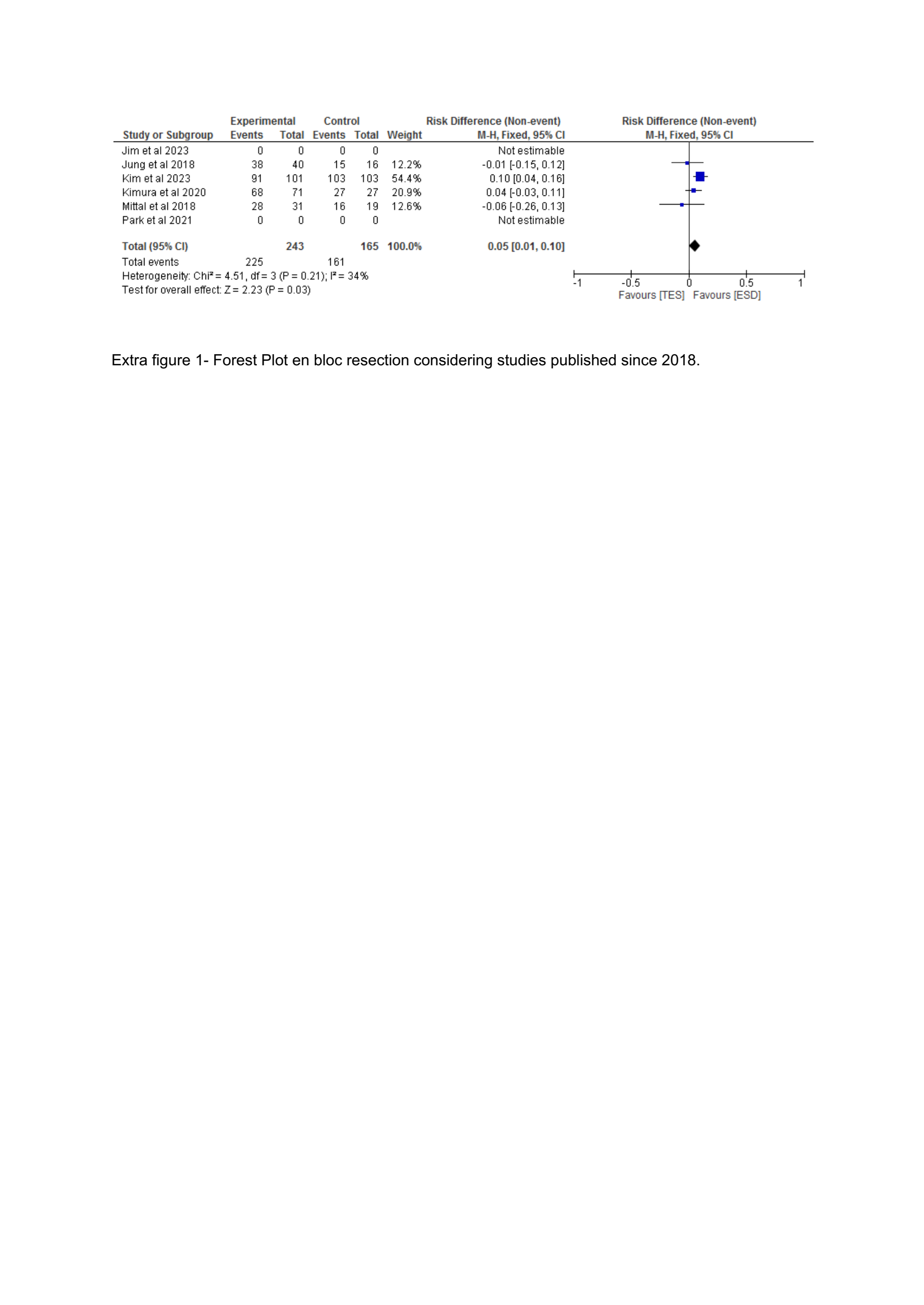

Supplement: Supplementary file 1 [file mmc1.docx]
